# Comparative Efficacy and Safety of Hybrid Endoscopic Submucosal Dissection for Colorectal Neoplasia: A Systematic Review and Meta‐Analysis

**DOI:** 10.1002/jgh3.70349

**Published:** 2026-03-05

**Authors:** Moaz Alowami, Chitrala Sruthi, Simona Sehrish, Ahmed Elamin, Lakshmish Devang Halepalya Somashekar, Salman Majeed, Shahid Ullah, Priyanka Deb Nath, Adeel Bin Tariq, Muhammad Yasir, Muhammad Aamir Shahzad, Khalifa Saleh Alteneiji, Rhoda Oluwatise Babasola, Tallal Hashmi, Muzammil Farhan, Syed Anjum Gardezi, Mumtaz Hayat

**Affiliations:** ^1^ Libyan International Medical University Benghazi Libya; ^2^ University Hospitals Coventry & Warwickshire Coventry UK; ^3^ Department of Gastroenterology Cardiff and Vale Health Board Cardiff UK; ^4^ Department of Gastroenterology Leicester Royal Infirmary Leicester UK; ^5^ Department of Medicine Northampton General Hospital Northampton UK; ^6^ Department of Medicine North Tees Hospital Stockton UK; ^7^ University Hospitals Plymouth NHS Trust Plymouth UK; ^8^ Department of Gastroenterology University Hospitals of Northamptonshire NHS Trust Northampton UK; ^9^ Department of Gastroenterology Sheikh Tahnoon Medical City Alain UAE; ^10^ Department of Medicine University of Sunderland Sunderland UK; ^11^ Department of Medicine Rawalpindi Medical University Rawalpindi Pakistan; ^12^ Imperial College London London UK; ^13^ Consultant Gastroenterologist & Advanced Endoscopist, Jhons Hopkins HealthCare Aramco Dhahran Kingdom of Saudi Arabia; ^14^ Department of Gastroenterology Northumbria Healthcare Newcastle upon Tyne UK

**Keywords:** colorectal cancer, endoscopic dissection, hybrid submuscosal dissection

## Abstract

**Background:**

Hybrid endoscopic submucosal dissection (h‐ESD) has emerged as a modified approach to overcome the technical challenges associated with conventional ESD (c‐ESD). However, evidence comparing their safety and efficacy in colorectal neoplasia remains limited.

**Methods:**

A comprehensive search was conducted in PubMed, Cochrane Library, and Embase up to April 2025 for randomized and propensity‐matched studies comparing h‐ESD with c‐ESD for colorectal neoplasia. The primary outcome was en bloc resection, with secondary outcomes including procedure time, adverse events, bleeding, and perforation. Data synthesis was performed using a random‐effects model in RevMan.

**Results:**

Five studies (three RCTs and two propensity‐matched cohorts) involving 1047 participants were included. The pooled analysis demonstrated no significant differences in en bloc resection rates (OR = 0.64, 95% CI = 0.26–1.56; *p* = 0.33; *I* ^2^ = 69%) or R0 resection rates (OR = 0.70, 95% CI = 0.44–1.11; *p* = 0.13; *I* ^2^ = 24%). h‐ESD was associated with significantly shorter procedure duration (WMD = −10.65 min, 95% CI: −14.90 to −6.39; *p* < 0.01; *I* ^2^ = 5%). No significant differences were observed for overall adverse events (OR = 1.14, 95% CI: 0.70–1.84), bleeding episodes (OR = 1.28, 95% CI: 0.45–3.65), or bowel perforation (OR = 0.97, 95% CI: 0.54–1.73).

**Conclusion:**

Hybrid ESD demonstrated equivalent safety and efficacy to c‐ESD for colorectal neoplasia, with the added advantage of significantly shorter procedure times. Further high‐quality RCTs are needed to validate its role in clinical practice.

## 1 Introduction

Colorectal cancer (CRC) remains a major global health burden, ranking as the third most frequently diagnosed malignancy and the second leading cause of cancer‐related deaths worldwide [1]. In 2012, it was responsible for an estimated 1.4 million new cases and nearly 700 000 deaths [2]. Projections indicate that by 2030, the incidence will rise by approximately 60%, surpassing 2.2 million new cases and 1.1 million deaths annually [3]. Central to the pathogenesis of the majority of these malignancies is the progression from precursor lesions. The precursor lesions are either pathologically classified as conventional adenomas or sessile serrated polyps [4]. Refined screening guidelines, effective imaging strategies, and robust interventional procedures are all integral to combating this cumulative burden on global morbidity and mortality. Endoscopic treatment offers a more minimally invasive approach to a more burdensome surgical resection.

Endoscopic submucosal dissection (ESD) is a minimally invasive technique designed for the removal of large polyps or early‐stage, non‐metastatic lesions [5]. Originating in Japan in the late 1990s, ESD was introduced to overcome the limitations of endoscopic mucosal resection (EMR) in managing extensive superficial gastric cancers [6]. The ESD procedure involves a circumferential mucosal incision followed by hypervigilant dissection of the submucosal layer with a specialized electrosurgical knife [7]. Conventional EMR employs a snare combined with electrocautery to excise the targeted tissue. However, when lesions exceed approximately 20 mm in size, EMR often necessitates removal in multiple fragments rather than en bloc. This leads to a higher risk for local cancer recurrence and reduced accurate histopathological staging quality. Unlike EMR, ESD enables the removal of lesions in a single piece. Although technically demanding, associated with prolonged procedure times, and carrying a higher risk of complications, ESD provides distinct advantages. These include superior histopathological evaluation of resection margins, reduced recurrence rates, and an enhanced likelihood of curative treatment through a minimally invasive approach [8, 9]. The widespread adoption of ESD in the United States has been impeded due to a lack of mentors, technical demands, longer procedure times, and concerns regarding adverse events [7].

The clinical application of conventional ESD (C‐ESD) in the colorectal anatomical proximity presents inherent technical challenges. The colon presents with an array of anatomical complexities, including a thin wall, pronounced haustral folds, and sharp flexures. This subsequently makes endoscopic maneuverability technically challenging to achieve. These factors contribute to substantially longer procedure times, a higher risk of adverse events such as perforation, and a notoriously steep learning curve for endoscopists and resident trainees. A new technique aims to challenge the inherent limitations of conventional ESD while enhancing its oncological advantages. Hybrid ESD combines an initial circumferential incision and partial submucosal dissection with a final snare‐based resection. Hybrid‐ESD biomechanically integrates the foundational elements of both EMR and conventional ESD. The bioengineered purpose of Hybrid‐ESD is to simplify the most technically demanding, time‐consuming, and highest risk portion of C‐ESD, which is the extensive dissection of the deep central submucosa. By doing so, H‐ESD aims to shorten procedure duration and improve safety while still achieving an en bloc specimen [10, 11].

A noteworthy paucity in existing literature of head‐to‐head comparative data exists for hybrid versus conventional ESD. Therefore, the primary objective of this systematic review and meta‐analysis is to comprehensively evaluate the comparative efficacy and safety of these two techniques for the endoscopic management of colorectal cancer. A robust qualitative and quantitative synthesis via statistical pooling of high‐quality data will provide valuable clinical insight into the efficacy and safety of the respective procedures.

## 2 Methods

This meta‐analysis was conducted in accordance with the PRISMA (Preferred Reporting Items for Systematic Reviews and Meta‐Analyses) reporting guidelines. As no primary patient data were involved, ethical approval was not required. The study protocol has been prospectively registered in PROSPERO (CRD420251082964).

### 2.1 Data Sources and Searches

A systematic literature search was conducted in PubMed, Embase, and the Cochrane databases, covering all records from inception through April 2025. To supplement the electronic search, reference lists of trials, observational studies, and review studies were manually screened to capture additional eligible studies. Search terms combined relevant Medical Subject Headings (MeSH) and keywords such as “Hybrid ESD,” “waterjet,” “submucosal dissection,” and “RCT,” with Boolean operators (AND, OR) used to refine retrieval (Table S1).

### 2.2 Eligibility Criteria

To be eligible for inclusion in this systematic review and meta‐analysis, studies were required to meet the following predefined criteria: (i) randomized controlled trials (RCTs) or propensity‐matched cohort studies evaluating the effects of hybrid and conventional endoscopic submucosal dissection, (ii) comparison of the intervention arm of H‐ESD and the comparator arm C‐ESD, (iii) reporting of at least one key clinical outcome was required, including en‐bloc resection rate, adverse events and procedural duration.

Studies were excluded if they fulfilled any of the following conditions: conducted in non‐human subjects; outside the scope of the review; published as reviews, editorials, letters, comments, or conference abstracts; case reports or small case series with fewer than 10 participants; or lacking sufficient information to assess the specified outcomes. Furthermore, non‐randomized or unmatched studies, investigations involving pediatric populations (< 18 years), trials without a comparator group, and studies addressing outcomes unrelated to procedural efficacy or safety were not considered.

### 2.3 Selection Process

We utilized Rayyan to manage the initial screening process and to remove duplicate records from the search results. Following deduplication, two authors independently reviewed all titles and abstracts. Subsequently, the same authors conducted a detailed full‐text assessment. Any disagreements that arose during this process were resolved through the involvement of a third author.

### 2.4 Data Extraction

Data from eligible studies were systematically retrieved with the aid of a standardized extraction template. The collected variables included the name of the first author, year of publication, total number of participants, baseline demographic characteristics (such as age and sex), associated comorbidities, pathological features, duration of follow‐up, and clinical outcomes reported. The primary outcomes were en‐bloc resection and R0 resection. Secondary outcomes included procedure duration, overall adverse events, bleeding episodes, and bowel perforation.

### 2.5 Risk of Bias Assessment

The quality of randomized controlled trials was evaluated with the Cochrane Risk of Bias 2.0 tool [12]. For observational studies, the ROBINS‐I was used [13]. RoBvis was used to create traffic plots and a summary of bias assessment in every domain.

### 2.6 Data Analysis

All statistical analyses were performed using RevMan (Cochrane Collaboration, Copenhagen, Denmark). Binary outcomes were pooled as odds ratios (ORs) with 95% confidence intervals (CIs), whereas continuous outcomes were reported as weighted mean differences (WMDs) with corresponding 95% CIs. To address potential variability among studies, a random‐effects model was applied. Statistical heterogeneity was evaluated using the *I* ^2^ statistic, with values of 50% or higher considered indicative of substantial heterogeneity. Because fewer than 10 studies were included, publication bias was not examined through funnel plot analysis or other formal statistical methods.

## 3 Results

### 3.1 Study Selection

**FIGURE 1:**
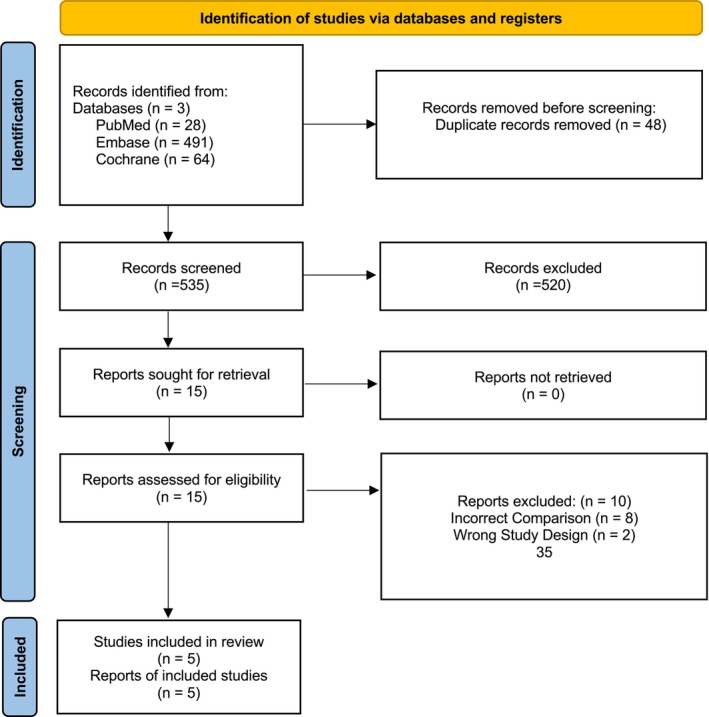
PRISMA flowchart depicting the screening and study selection process.

A total of 583 studies were initially identified across various databases. After removing 48 duplicate entries, 535 studies remained. These were screened based on their titles and abstracts, following which five studies met the eligibility criteria and were included in the final analysis (Figure 1).

### 3.2 Study Characteristics

A total of five studies, comprising 1047 participants, were included in the analysis. These consisted of three randomized controlled trials [14, 15, 16] and two propensity score–matched cohort studies [17, 18]. Across RCTs and cohort studies, h‐ESD and c‐ESD groups were well balanced in baseline characteristics (age, sex, lesion type, and size). Adenomas were the most common pathology, with limited representation of invasive cancers. Detailed study characteristics are reported in Table 1. The bias assessment showed that most studies were judged to have a low to moderate risk of bias (Figures S1 and S2).

**TABLE 1:** Baseline Characteristics of included studies.

Author year	Sample size		Study design	Age in years		Male (%)		Pathology findings
	h‐ESD	c‐ESD		h‐ESD	c‐ESD	h‐ESD	c‐ESD	
Bae 2015	34	31	RCT	63.0 (44–82)	66.0 (37–79)	58.8	58.1	In both groups, most lesions were adenomas, followed by intramucosal cancers, with few superficial or deep submucosal cancers.
Morimonto 2023	100	100	Cohort study	69 ± 11	68 ± 11	55	62	In both C‐ESD and SH‐ESD groups, adenomas were most common, followed by Tis carcinoma, with fewer T1a and T1b carcinomas.
Yang 2024	40	49	RCT	62.7 ± 9.8	61.6 ± 10.9	50	51.1	In both H‐ESD and C‐ESD groups, the most common morphology was laterally spreading granular or nongranular tumors, predominantly classified as Paris IIa lesions.
Zhang 2024	263	263	Cohort study	64.5 ± 10.7	64.4 ± 10.9	54.8	53.2	In both planned H‐ESD and C‐ESD groups (after matching), adenomas were most frequent, followed by carcinomas and sessile serrated lesions, with most tumors confined to the mucosa and few showing submucosal invasion.
Zhou 2015	40	38	RCT	64.3 ± 9		NR		In both C‐ESD and HK‐ESD groups, lesions were predominantly large colorectal neoplasms without deep submucosal invasion, with no significant difference in patient demographics, lesion location, or size.

Abbreviations: c‐ESD, conventional endoscopic submucosal dissection; C‐ESD, conventional endoscopic submucosal dissection; h‐ESD, hybrid endoscopic submucosal dissection; H‐ESD, hybrid endoscopic submucosal dissection; HK‐ESD, hook knife endoscopic submucosal dissection; NR, not reported; RCT, randomized controlled trial; SH‐ESD, simplified hybrid endoscopic submucosal dissection; T1a, tumor invades submucosa ≤ 1 mm; T1b, tumor invades submucosa > 1 mm; Tis, carcinoma in situ.

### 3.3 Primary Outcome

#### 3.3.1 En‐Bloc Resection

**FIGURE 2:**
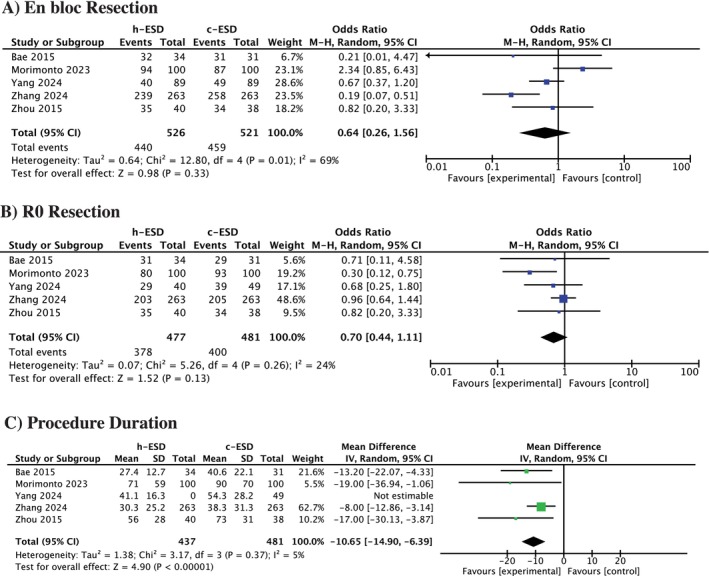
Forest plot for (A) En‐bloc resection, (B) R0 resection, (C) Procedure duration.

All five studies provided data on en bloc resection rates. The pooled analysis indicated no significant difference between h‐ESD and c‐ESD (OR = 0.64, 95% CI = 0.26 to 1.56; *p* = 0.33, *I* ^2^ = 69%) (Figure 2A).

#### 3.3.2 R0 Resection

All five studies reported on R0 resection. The pooled analysis indicated no significant difference between h‐ESD and c‐ESD (OR = 0.70, 95% CI = 0.44 to 1.11; *p* = 0.13, *I* ^2^ = 24%) (Figure 2B).

### 3.4 Secondary Outcomes

#### 3.4.1 Procedure Duration

All five studies reported procedure duration. The pooled analysis demonstrated that h‐ESD was associated with a significantly shorter procedure time compared with c‐ESD (WMD = −10.65 min, 95% CI = −14.90 to −6.39; *p* < 0.01, *I* ^2^ = 5%) (Figure 2C).

#### 3.4.2 Overall Adverse Events

**FIGURE 3:**
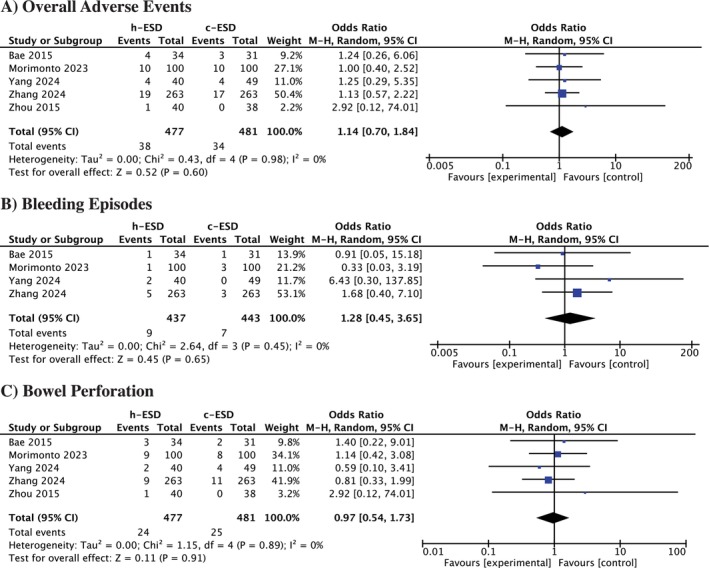
Forest plot for (A) Overall adverse events, (B) Bleeding episodes, (C) Bowel perforation.

Adverse events reported included electrocoagulation syndrome, bleeding, and bowel perforation. The pooled analysis showed no significant difference between h‐ESD and c‐ESD (OR = 1.14, 95% CI = 0.70 to 1.84; *p* = 0.60, *I* ^2^ = 0%) (Figure 3A).

#### 3.4.3 Bleeding Episodes

The pooled analysis for bleeding episodes demonstrated no significant difference between the two groups (OR = 1.28, 95% CI = 0.45 to 3.65; *p* = 0.65, *I* ^2^ = 0%) (Figure 3B).

#### 3.4.4 Bowel Perforation

Regarding bowel perforation, the pooled analysis demonstrated no significant difference between the two groups (OR = 0.97, 95% CI = 0.54 to 1.73; *p* = 0.91, *I* ^2^ = 0%) (Figure 3C).

## 4 Discussion

This systematic review and meta‐analysis compared hybrid and conventional endoscopic submucosal dissection for colorectal neoplasia. Five eligible studies comprising a total of 1047 participants were included in the pooled analysis. Across the primary and secondary outcomes assessed—including en bloc resection, R0 resection, adverse events, bleeding, and bowel perforation—no significant differences were observed between the two approaches. Notably, H‐ESD was associated with a significantly shorter procedure duration compared to C‐ESD, reflecting a potential procedural advantage. Overall, the findings suggest that while H‐ESD may offer efficiency benefits, its efficacy and safety outcomes remain comparable to those of C‐ESD.

Our findings are consistent with the recent study by Zhang et al., which enrolled 526 patients with equal allocation through propensity score matching [17]. This study reinforced the procedural efficiency of H‐ESD, demonstrating significantly reduced operative duration compared with C‐ESD. However, it also highlighted that H‐ESD achieved a slightly lower en bloc resection rate than C‐ESD for lesions ≥ 40 mm. Similarly, Yang et al. emphasized the importance of procedural efficiency, reporting shorter procedural times with H‐ESD [14]. The remaining included studies echoed these observations, consistently supporting the superior efficiency of the hybrid technique.

Procedural duration is clinically significant, as longer endoscopic times increase risks such as perforation, post‐polypectomy syndrome, and anesthesia‐related complications [19]. Several studies, including large Japanese and European cohorts, have emphasized that shorter procedures improve patient tolerance and resource utilization [20]. By substantially reducing operative time, H‐ESD may contribute to better patient outcomes, lower intraprocedural risks, and improved adoption by less experienced endoscopists. This has important implications for training, as conventional ESD has a steep learning curve and limited uptake outside Asia [21]. Our analysis demonstrated comparable adverse events between both approaches, suggesting that the hybrid technique may enhance efficacy without compromising safety.

H‐ESD represents an important advancement in the minimally invasive treatment of colorectal neoplasia. By integrating the precision of ESD with the efficiency of EMR, h‐ESD enables high en bloc resection rates for complex lesions that previously would have required piecemeal EMR. This provides intact specimens for histopathological evaluation, allowing for more accurate assessment of resection margins and depth of invasion [10]. Importantly, the shorter procedural duration associated with h‐ESD reduces intraprocedural risks, particularly perforation, while maintaining oncological safety. However, our analysis demonstrated comparable en bloc and R0 resection rates between hybrid and conventional ESD. Compared with conventional ESD, h‐ESD is faster, less technically demanding, and potentially safer, making it an attractive option for endoscopists and interventionalists [11]. Operator experience is also known to influence ESD outcomes, with higher expertise associated with improved en bloc and R0 resection rates and fewer complications. Lesion characteristics also play a role, as non‐granular laterally spreading tumors and proximal colonic lesions are technically more challenging and carry higher risks of incomplete resection and perforation compared with granular and rectal lesions. Future cutting‐edge research in the form of high‐quality prospective randomized controlled trials will allow for a more comprehensive analytical synthesis of contemporary evidence.

Strengths in this meta‐analysis include a comprehensive search strategy that was devised to improve sensitivity and specificity in a research domain of limited literature. The search across multiple known academic electronic databases from their inception allows for effective retrieval of peer‐reviewed articles. The inclusion of randomized controlled trials and propensity‐matched observational data allows for a robust evidence base. Strict inclusion and exclusion criteria, established by a physician with a professional background in the topic, ensured clinical relevance. Noteworthy limitations included the limited number of contemporary studies available. The total number of participants in the limited pooled analysis is also relatively small for a meta‐analysis. Additionally, heterogeneity in the definitions and reporting of outcomes across studies represents a potential source of bias. Limited outcomes were reported among the studies included, which limited a more comprehensive statistical pooling of other potential outcomes. Since there were fewer than 10 studies eligible, publication bias assessment was not feasible.

## 5 Conclusion

In summary, this meta‐analysis demonstrates that hybrid endoscopic submucosal dissection offers comparable safety and efficacy to conventional ESD in the management of colorectal neoplasms, with the added advantage of significantly shorter procedural duration. While en bloc and R0 resection rates and adverse event profiles were similar between the two techniques, the procedural efficiency of H‐ESD highlights its potential as a practical and less technically demanding alternative. These findings underscore the promise of H‐ESD as an evolving therapeutic approach; however, further large‐scale, high‐quality randomized controlled trials are needed to validate these outcomes and to define its role within standard clinical practice.

## Funding

The authors have nothing to report.

## Consent

The authors have nothing to report.

## Conflicts of Interest

The authors declare no conflicts of interest.

## Supporting information


**Table S1:** Search strategy.
**Figure S1:** Bias assessment for RCTs.
**Figure S2:** Bias assessment for observational studies.

## Data Availability

The data that support the findings of this study are available from the corresponding author upon reasonable request.
