# Supplementary material for: Comparative Efficacy and Safety of Hybrid Endoscopic Submucosal Dissection for Colorectal Neoplasia: A Systematic Review and Meta‐Analysis
Source: JGH Open. 2026 Mar 5;10(3):e70349. doi: 10.1002/jgh3.70349 (PMC12963464; doi:10.1002/jgh3.70349)
Supplement: Supplementary file 1 — Table S1: Search strategy. Figure S1: Bias assessment for RCTs. Figure S2: Bias assessment for observational studies. [file JGH3-10-e70349-s001.docx]

**Table S1: Search strategy**

| Database | Search string | Results |
| --- | --- | --- |
| PubMed | ("Hybrid ESD" OR "hybrid technique" OR "hybrid endoscopic submucosal dissection" OR "HybridKnife" OR "waterjet" OR "KH-ESD" OR "snare-assisted ESD") AND ("Endoscopic Submucosal Dissection"[Mesh] OR "endoscopic submucosal dissection" OR "ESD" OR "conventional ESD" OR "standard ESD") AND ("Colorectal Neoplasms"[Mesh] OR "colorectal lesion*" OR "colorectal tumor*" OR "colorectal polyp*" OR "colorectal neoplasia" OR "colon" OR "rectal" OR "Cancer" OR "neoplasms") AND ("randomized controlled trial" OR "RCT" OR "prospective study" OR "propensity score matching" OR "propensity-matched" OR "Randomized" OR "Randomised" OR "matched" OR "trial") | 28 |
| Cochrane | ( Hybrid ESD OR hybrid technique OR hybrid endoscopic submucosal dissection OR HybridKnife OR waterjet OR KH ESD OR snare assisted ESD ) AND ( endoscopic submucosal dissection OR ESD OR conventional ESD OR standard ESD ) AND ( colorectal lesion* OR colorectal tumor* OR colorectal polyp* OR colorectal neoplasia OR colon OR rectal OR cancer OR neoplasms ) AND ( randomized controlled trial OR RCT OR prospective study OR propensity score matching OR propensity matched OR randomized OR randomised OR matched OR trial ) | 64 |
| Embase | ( Hybrid ESD OR hybrid technique OR hybrid endoscopic submucosal dissection OR HybridKnife OR waterjet OR KH ESD OR snare assisted ESD ) AND ( endoscopic submucosal dissection OR ESD OR conventional ESD OR standard ESD ) AND ( colorectal lesion* OR colorectal tumor* OR colorectal polyp* OR colorectal neoplasia OR colon OR rectal OR cancer OR neoplasms ) AND ( randomized controlled trial OR RCT OR prospective study OR propensity score matching OR propensity matched OR randomized OR randomised OR matched OR trial ) | 491 |

**Figure S1: Bias assessment for RCTs**

**
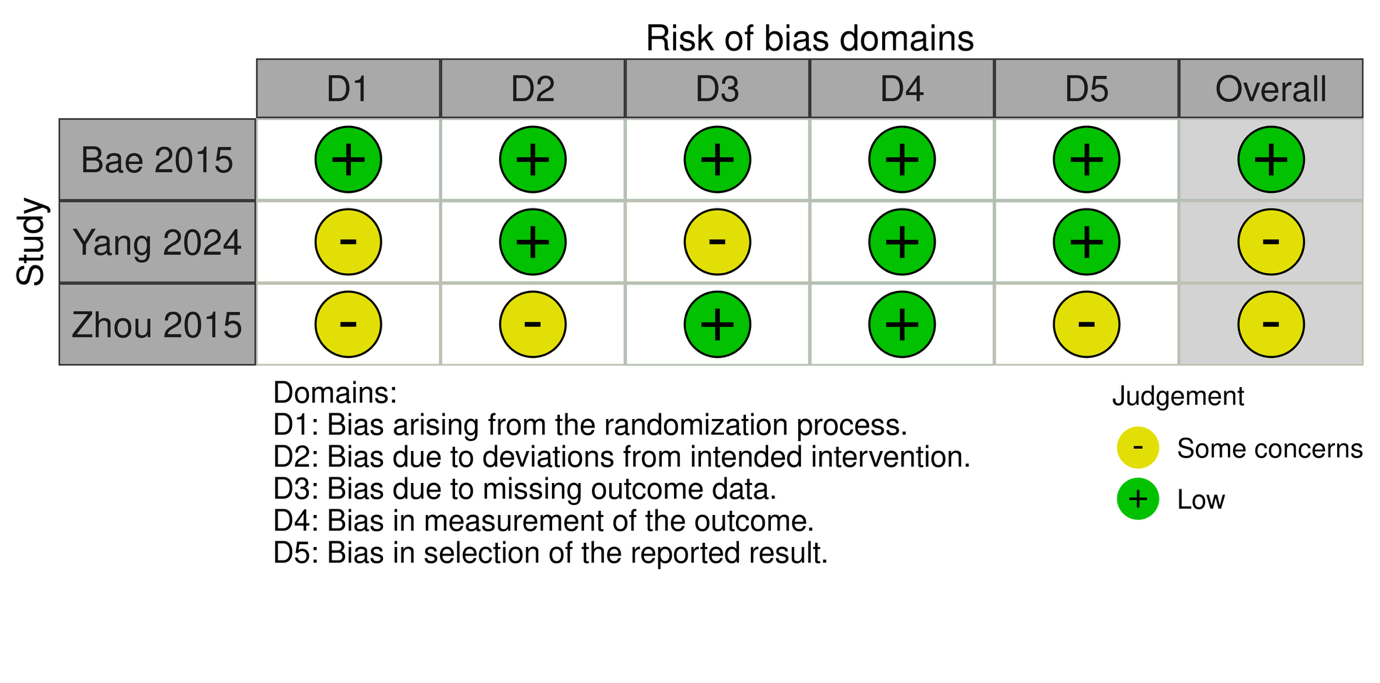
**

**Figure S2: Bias assessment for observational studies**

**
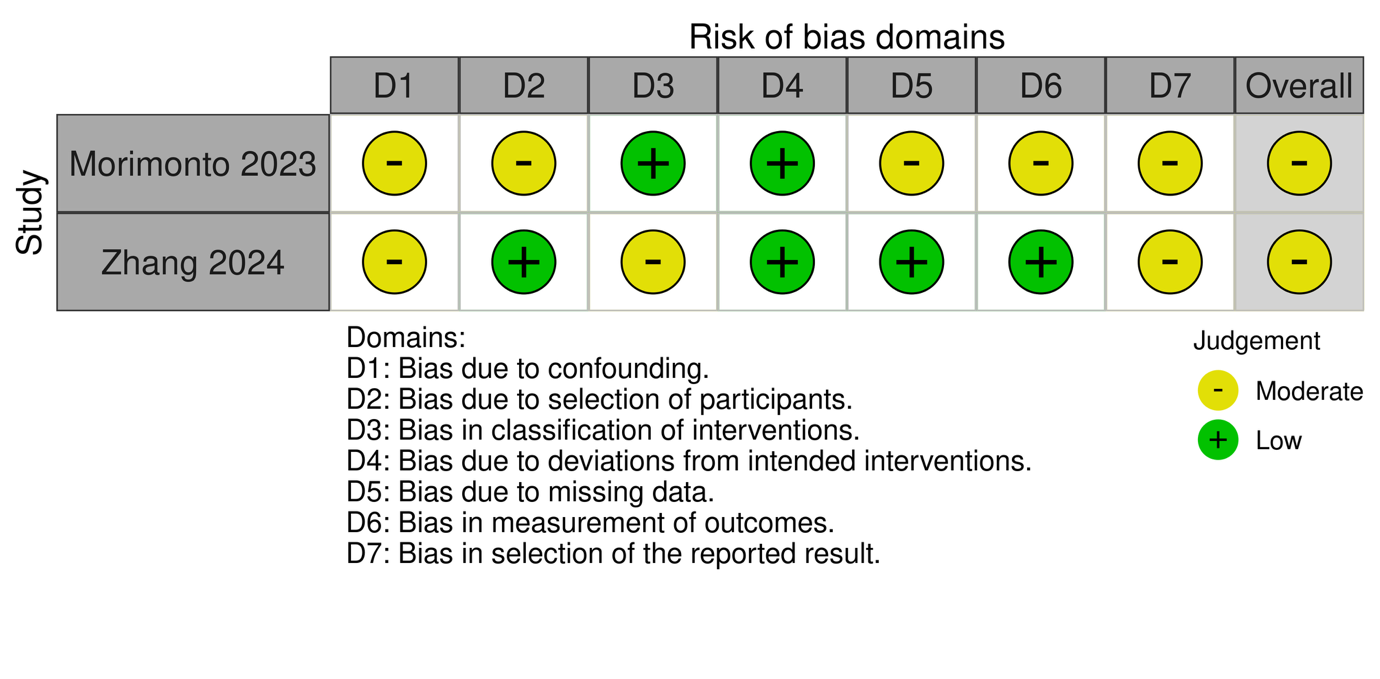
**
